# Patterns of GPS Tracks Suggest Nocturnal Foraging by Incubating Peruvian Pelicans (*Pelecanus thagus*)

**DOI:** 10.1371/journal.pone.0019966

**Published:** 2011-05-25

**Authors:** Carlos B. Zavalaga, Giacomo Dell'Omo, Paolo Becciu, Ken Yoda

**Affiliations:** 1 Graduate School of Environmental Studies, Nagoya University, Nagoya, Japan; 2 Ornis italica, Rome, Italy; Institut Pluridisciplinaire Hubert Curien, France

## Abstract

Most seabirds are diurnal foragers, but some species may also feed at night. In Peruvian pelicans (*Pelecanus thagus*), the evidence for nocturnal foraging is sparse and anecdotal. We used GPS-dataloggers on five incubating Peruvian pelicans from Isla Lobos de Tierra, Perú, to examine their nocturnality, foraging movements and activities patterns at sea. All instrumented pelicans undertook nocturnal trips during a 5–7 day tracking period. Eighty-seven percent of these trips (n = 13) were strictly nocturnal, whereas the remaining occurred during the day and night. Most birds departed from the island after sunset and returned a few hours after sunrise. Birds traveled south of the island for single-day trips at a maximum range of 82.8 km. Overall, 22% of the tracking period was spent at sea, whereas the remaining time was spent on the island. In the intermediate section of the trip (between inbound and outbound commutes), birds spent 77% of the trip time in floating bouts interspersed by short flying bouts, the former being on average three times longer than the latter. Taken together, the high sinuosity of the bird's tracks during floating bouts, the exclusively nocturnal trips of most individuals, and the fact that all birds returned to the island within a few hours after sunrise suggest that pelicans were actively feeding at night. The nocturnal foraging strategy of Peruvian pelicans may reduce food competition with the sympatric and strictly diurnal Guanay cormorants (*Phalacrocorax bougainvillii*), Peruvian boobies (*Sula variegata*) and Blue-footed boobies (*S. nebouxii*), which were present on the island in large numbers. Likewise, plankton bioluminescence might be used by pelicans as indirect cues to locate anchovies during their upward migration at night. The foraging success of pelicans at night may be enhanced by seizing prey close to the sea surface using a sit-and-wait strategy.

## Introduction

Most seabirds are visual predators whose foraging is constrained by the duration of daylight [1]. However, some species are active at night in a facultative or condition-dependent way apparently to reduce interference competition with other seabirds [2], to take advantage of the diel vertical migration of prey [3], to avoid predators [4], [5], and/or to obtain fish discards and bait during nocturnal fishing operations [6], [7]. In the case of pelicans, direct observations of foraging birds and telemetry studies have shown that five out of the eight extant species of pelicans may facultatively forage at night (see review in Nelson (1985) [8]). Nocturnal habit appears to be opportunistic in Brown pelicans (*Pelecanus occidentalis*) [9], and common in American white pelicans (*P. erythrorhynchos*) but associated with a lower prey capture rate compared to daytime foraging [10]. However, the empirical data on these species are limited and the adaptive advantages of nocturnal foraging for pelicans are unclear.

Peruvian pelicans (*P. thagus*) are resident seabirds of the Humboldt Current upwelling system, breeding from northern Perú to central Chile [11], [12]. Unlike all other member of the Pelecanidae, Peruvian pelicans are strict marine predators consuming primarily Peruvian anchovies (*Engraulis ringens*) and other pelagic fish [13], [14], [15] that are captured by shallow plunge-diving, surface-seizing or through kleptoparasitism [16]. Additionally, they efficiently scavenge fish discards and offal at fishing ports and boats (Zavalaga C.B., pers. observ.). Based on observations of birds returning to the colony a few hours following sunset, and at every hour during full moon nights, some have hypothesized that Peruvian pelicans engage in nocturnal foraging activities [17], [18]. Nevertheless, to date no studies are available to validate this assumption, and it is still unknown whether Peruvian pelicans actively feed at night or are merely commuting during the night after foraging during the daytime.

Here, we provide the first snapshot of Peruvian pelican foraging movements and activities patterns at sea, and demonstrate that they undertake nocturnal foraging trips after deploying GPS dataloggers on five incubating birds from Isla Lobos de Tierra, Perú. We subsequently evaluate possible causes and adaptive advantages of nocturnal foraging.

## Materials and Methods

Incubating Peruvian pelicans were studied at Isla Lobos de Tierra (6°24′S, 80°51′W), Perú between 15 and 22 December 2010. Lobos de Tierra is a barren island with an estimated area of 1426 ha (9 km max. length, 3 km max. width). All pelicans on Lobos de Tierra were found at early stages of the breeding cycle, with most birds building nests or incubating eggs. They occupied the large desert plains and beaches of the northern side of the island, nesting in several clusters of different sizes (100s and 1000s of nests). From a visual inspection of photographs of the entire pelican colony, we estimated a breeding population size of 160,000–210,000 pairs during the study period. Wind blew predominantly from the southeast (circular mean  = 183±16°) at a mean speed of 16.9±4.4 km•h^−1^ (hourly wind measurements were obtained between 15 and 22 December from the meteorological station of the Peruvian Navy Hydrographic Service located at Isla Lobos de Afuera, 65 km south of Lobos de Tierra).

### Capture and handling

Peruvian pelicans are very timid, leaving their eggs unattended when researchers approach their nests. To avoid egg predation by aerial predators we selected birds from different breeding clusters (∼ 50–100 nests each) that were located close to the guards' houses where the presence of potential predators was reduced. To capture incubating pelicans, we crawled toward peripheral nests and placed a thin-thread noose loop on top of the nest containing a clutch of three eggs; we then retreated 10–15 m away. Pelicans returned to their nests and resumed incubation 3–5 min after the trap was set up. The noose was triggered remotely after the pelican seated on the nest. Once the noose was closed, we slowly pulled the pelican away from the breeding cluster, producing little disturbance on the neighbor incubating pelicans. All prey items regurgitated during captures were identified. After 5 minutes of handling and GPS attachment, the bird's crown and neck were marked with red dye (Rhodamine B) for easy identification at distance. Pelicans were released close to their breeding cluster and we watched the unattended eggs until tagged birds resumed incubation (no longer than 10 minutes).

### GPS dataloggers

Five incubating pelicans were fitted with GiPSy-2 GPS dataloggers (Technosmart s.r.l. www.technosmart.eu) programmed to record either one fix every second for three loggers or one at 10 sec intervals for two loggers. We anticipated a difficult recapture and possible loss of the GPS given the nervous nature of Peruvian pelicans. Thus, to recover GPS records without recapturing birds, we added a blue-tooth board (BT, 1.5 g) to the GPS to allow remote data downloading and memory resetting at distance. An external USB - BT antenna adapter was used to optimize communication between a portable computer and the GPS at distances >20 m. Each GPS was powered with three LS 14500 SAFT batteries (2600 mAh, 3.7 V) connected in parallel. The entire assemblage was encapsulated in heat-shrink plastic tubing and attached to the bird's lower back feathers (above the uropygeal gland) with waterproof Tesa tape®. The GPS and accessories had a combined weight of 90 g, which represented <1.8% of the adult's weight (5–7 kg, [11]). This percentage is below the accepted <3% of device to body mass threshold for causing adverse behavioral effects [19]. The loggers would fall off with the feathers before or at molt. The tagged birds were searched from a distance with the aid of binoculars twice per day (10:00–11:00 h, and 16:00–17:00 h) to verify their presence around the breeding clusters. Although GPS signals were detected at distances >20 m, we obtained uninterrupted data downloading at closer range (usually 8–10 m from the nest) without disrupting the normal pelican activities.

Four out of five pelicans resumed their breeding duties after attachment of the loggers and were seen incubating their eggs by the time we left the island. We were able to download complete data from all these birds. One bird was seen 24 h after capture and was not resighted again; but the mate was still incubating by the time we left the island. We were able to download data from this bird for the first 24 h, which included the record of an incomplete foraging trip. Because the inbound path was truncated 5 km from the island, the initial data of the fifth bird was included only for the calculations of maximum foraging distance, departure time and at-sea activities.

### Data analysis

**Figure 1:**
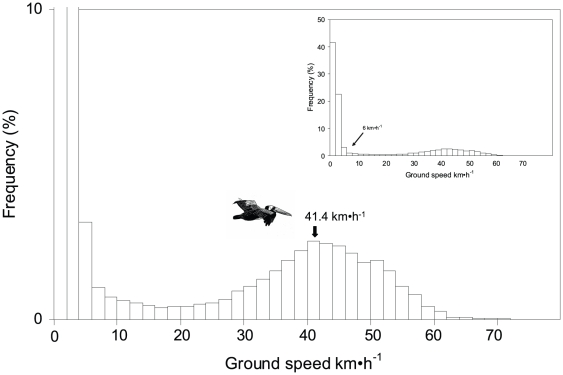
Frequency distribution of ground speed between consecutive GPS locations of incubating Peruvian pelicans. The inset graph shows the cut-off value to discriminate flying speeds from floating on the water speeds. The outer distribution depicts the mean flight speed.

**Figure 2:**
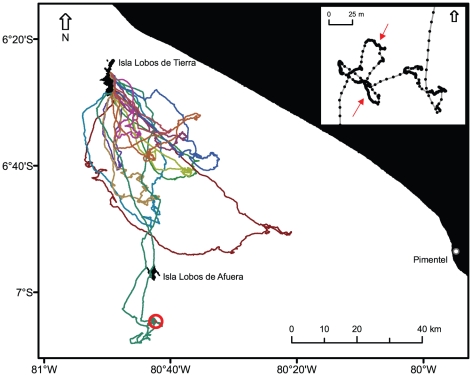
Foraging GPS-tracks of five incubating Peruvian pelicans from Isla Lobos de Tierra. Individual trips are represented by different colors. The inset map shows a zoomed-in portion of one track (indicated by the red circle). Floating bouts are shown by darker paths (two bouts indicated by red arrows) and flying bouts by more interspersed position fixes joined by lines.

The spatial data from loggers were mapped and analyzed using ArcGIS 9.2 Geographic Information System (ESRI Inc., Redlands, CA). The positions were projected on the UTM coordinate system (Zone 14S) for all spatial analysis. The high resolution (<10 m in >95% of locations after excluding fixes with DOP values >6, GiPSy-2 user's manual, www.technosmart.eu) and short recording intervals of the loggers allowed us to identify the precise time budget of pelicans at sea. GPS data showed that after a nest shift, pelicans spent a variable amount of time on different activities away from the nest before departing for a trip. Likewise, some birds did not return to the nest immediately after completing a trip (see results), and consequently, for more accurate estimates, we defined a trip as the time elapsed between the departure from and arrival to the island. Instantaneous flight speeds were calculated from the distance and time between two consecutive GPS locations after excluding all points on land. An inspection of the frequency distribution of speeds revealed a discontinuity in movement patterns associated with speeds >6 km•h^−1^ (Fig. 1). We used this value to calculate the proportion of time within a trip when the bird was floating on the water (<6 km•h^−1^) and consequently, the proportion of time spent flying (> 6 km•h^−1^). This cut-off value has been reported as a typical pelican surface drifting speed [8]. Trips were divided in three sections:

- Outbound commute: from departure of the island to the location of the first landing on the water;
- Intermediate commute: from the first landing on the water to the last take-off on the water before returning to the island. This section was characterized by successive “floating bouts” defined as the time elapsed between landing on the sea surface and the next take-off (speeds <6 km•h^−1^, Fig. 2), and “flying bouts” defined as the time elapsed between the take-off to the next landing bout on the water (speeds >6 km•h^−1^, Fig. 2);
- Inbound commute: from the take-off location of the last floating bout to the first landing on the island.

For each section, we determined an index of path sinuosity defined as the ratio of the total distance traveled in a 30-sec interval to the straight line distance in that interval. This value was selected because longer intervals would have excluded a significant number of flying and floating bouts (see results). A sinuosity index close to 1 indicates high path linearity (probably associated with traveling), whereas high sinuosity values probably indicate food searches. The extent of the maximum potential foraging area in each trip was calculated in ArcGIS using the Minimum Convex Polygon estimation based on all points recorded during the intermediate commute. All statistical tests were performed using Statistical Analysis Systems (SAS Institute, 2004). Means were expressed ±1 s.d. when data were normally distributed, whereas medians were calculated when the data were highly skewed. Statistical differences were considered to be significant at α = 0.05.

## Results

At capture, all tagged pelicans regurgitated Peruvian anchovies. The availability of anchovies for pelicans around Lobos de Tierra during the study period was confirmed by the presence of this fish in 97% (n = 34) of regurgitations of sympatric incubating Peruvian boobies (*Sula variegata*) and Blue-footed boobies (*S. nebouxii*) that were also instrumented with data loggers as part of other fieldwork (Zavalaga et al., unpub. data). Overall, pelicans spent 78% of the tracking period (mean  = 5.5±1.05 days, range  = 4.4–6.8 days, n = 12 trips) on the island, the remaining time at sea. During 50% of at-sea trips (n = 12), pelicans landed 6–6.5 km south of the nesting site, remaining at the landing spot for several minutes (mean  = 69±22 min, range  = 37–92 min, n = 5) before returning to the nest. Some individuals undertook two consecutive trips without a nest changeover. Nest relief usually occurred in the morning (73% of shifts occurred between 07:00 and 10:30 h); however, pelicans did not immediately depart for a trip after being relieved at the nest. Incubation shifts averaged 13.8±7.78 h (range  = 5.1–26 h, n = 11).

All tracks of incubating Peruvian pelicans were oriented to the southeast and south of Isla Lobos de Tierra (mean vector bearing μ = 144°, vector length r = 0.95, Fig. 2). Mean trip duration was 11.2±5.5 h (Table 1), attaining a mean maximum range of 41.4±18.2 km and a mean total distance traveled of 151.5±73.6 km (Table 1). Flight speed averaged 41.4±1.5 km•h^−1^ (burst speed  = 80 km•h^−1^), but birds flew significantly faster with tail or cross-tail winds during inbounds (mean  = 50.4±2.8 km•h^−1^) than with head or head-tail winds during the outbound paths (mean  = 39.98±3.1 km•h^−1^, paired t-test, t_11_ = 6.83, P<0.001).

**Table 1:** Foraging variables of incubating Peruvian pelicans (*Pelecanus thagus*) from isla Lobos de Tierra, Perú, instrumented with GPS dataloggers.

Bird	Trip	Departure	Arrival	Period	Trip length (h)	Max. Distance (km)	Total Distance Traveled (km)	Destination Bearing (°)	% of the trip time floating on the water
1	1	12/16 21:12	12/17 05:28	Night	8.26	21.07	79.15	155	77
	2	12/17 19:39	12/18 06:40	Night	11.02	27.25	144.54	113	70
	3	12/19 00:08	12/19 06:23	Night	6.24	24.99	64.49	132	80
	4	12/20 17:04	12/21 06:32	Night	13.46	42.63	144.48	129	74
2	1	12/16 17:34	12/17 07:16	Night	13.70	82.82	278	172	50
	2	12/18 20:30	12/19 06:12	Night	9.69	39.82	129.68	140	77
3	1	12/17 18:30	12/18 07:07	Night	12.63	46.48	159.69	163	77
	2	12/19 10:13	12/20 06:21	Day/Night	20.13	41.95	207.47	162	81
	3	12/21 18:47	12/22 06:10	Night	11.37	30.02	136.75	130	78
4	1	12/19 02:14	12/19 06:29	Night	4.24	40.34	101.46	137	54
	2	12/20 04:03	12/20 07:16	Night	3.22	29.48	81.64	174	19
	3	12/21 10:21	12/22 07:19	Day/Night	20.98	73.84	290.45	130	77
5*	1	12/18 20:37	12/18<10:00	Night	-------	37.77	-------	138	-------

*Truncated approximately 5 km from the island during the inbound path.

All tagged pelicans undertook nocturnal trips away from the breeding colony. In two out of 12 excursions, at-sea activities were also recorded during daylight hours (Table 1), but even on these diurnal trips birds remained at sea overnight and returned to the island the next morning. Pelicans generally departed after sunset between 18:30–04:03 h, except for the two aforementioned trips. Arrivals to the island were more synchronous than departures and always occurred in the early morning (05:30–07:20 h). Pelicans spent a large proportion of the trip time on the intermediate commute (median  = 76.6%), which was divided into several floating and flying bouts (number of flying/floating bouts  = 9–141 per trip). Overall, 82% of these bouts took place between nautical sunset twilight (19:20 h) and nautical sunrise twilight (05:16 h). Floating bouts lasted longer (median  = 2.08 min, range  = 10 sec and 4.72 hrs) and were more frequent than flying bouts (median  = 43 sec, range  = 11 sec and 54.3 min; Kolmogorov-Smirnov test, D = 0.355, P<0.0001). The median total distance traveled in a trip during the intermediate commute (floating and flying bouts) was 69.71 km (range  = 8.5–196 km), covering a median area of 119 km^2^ (range  = 3–1604 km^2^).

**Figure 3:**
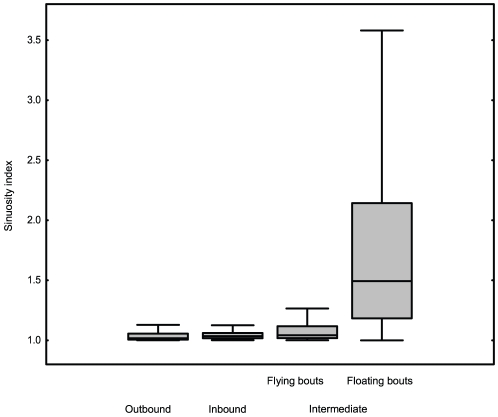
Sinuosity index during the beginning (outbound path), middle (food search) and ending (inbound path) stages of the trip. Box plots depict the 10, 25, 50, 75 and 90 percentiles of the distribution. A sinuosity index close to one means high path linearity.

The sinuosity index of the tracks during floating bouts was significantly higher than those during inbound and outbound flights, and during flying bouts that connected two consecutive landings on the water (REML, F_3,12_ = 16.4, P = 0.0002, Ryan's Q-test for pairwise comparisons, Fig. 3). Thus, these periods on the water surface were probably related to foraging. When the two trips with records during day/night hours were partitioned into two sections, we found that the proportion of the total floating time was higher at night (95% of the time from nautical sunset twilight to arrival on the island) than during the day (65% of the time from island departure to nautical sunset twilight). Likewise, the sinuosity index was higher (t-test, t = 4.6, P<0.0001) and the duration of floating bouts longer (t-test, t = 2.45, P  = 0.015) during nighttime.

## Discussion

This study reports the first description of the foraging behavior for Peruvian pelicans using GPS loggers, and, to our knowledge, it also represents the first attempt to track with precision the foraging trips of any member of the Pelecanidae. However, because we tracked a small number of birds (5 birds with a total of 13 trips) during a short period (5–7 days), the results must be taken with caution. Despite these limitations, the high sampling rate and accuracy of the instruments allowed the identification of detailed patterns of the activities at sea, previously undescribed in Peruvian pelicans.

It is clear from this study that all tagged birds undertook nocturnal trips. Diurnal trips were also observed in two birds that left the colony by mid-morning but during these excursions they stayed at sea also overnight, returning to the island on the next morning. Peruvian pelicans generally departed from the colony from late afternoon onwards and returned the next day within one hour after sunrise. Likewise, the sinuosity of the bird's tracks while floating on the water was much higher than during other sections of the trip (i.e. the birds were not passively drifting with the ocean currents, Fig. 2). All these results suggest that pelicans were probably feeding during these nocturnal excursions.

One plausible hypothesis for nocturnal foraging behavior may be temporal segregation with other species to mitigate inter-specific competition [2], [4], [20]. On Isla Lobos de Tierra, Peruvian pelicans breed sympatrically with Blue-footed and Peruvian boobies (combined population size estimated during the study period of approximately 200,000–300,000 birds, Zavalaga C.B., pers. observ.). Although Guanay cormorants (*Phalacrocorax bougainvillii*) do not breed on the island; they were present in large numbers during the study period (30,000 – 50,000 birds, Zavalaga C.B. pers. observ.). All of these species are significant predators of Peruvian anchovies [14], [21], [22]; however cormorants and boobies forage only during daytime [23], [24], [25] generally in large multi-species feeding flocks that also include pelicans and other species of seabirds [23], [26]. During the day, Brown and Peruvian pelicans primarily plunge-dive for access to their prey [27], [28], [29], but the well-developed subcutaneous air mattress prevents pelicans to dive more than partly below the surface [30]. Thus, the diving capabilities of Peruvian pelicans (suggested max. dive depth  = 2 m, [16]) are more limited than those of their counterparts in multi-species feeding flocks [24], [31], [32]. Given these differences, it is likely that pelicans capture bait fish driven to the surface by other seabirds or steal food from more proficient divers [16]. Nevertheless, the chances of successful foraging by pelicans in these flocks may be considerably reduced given the large number of intra- and inter-specific competitors in the vicinity of the breeding colony.

A second nonmutually exclusive hypothesis for the observed nocturnal foraging of pelicans may be related to the diel vertical movements of their prey [33]. There is ample body of evidence showing that Peruvian anchovies exhibit a full diel vertical migration, forming dense schools in deeper water strata during the day but scattering close to the surface by night (e.g. [34], [35]). This 24-h dark/light cycle may provide pelicans with a predictable source of food that can be exploited at night relatively close to the sea surface. The scattered horizontal distribution of anchovies at night probably requires pelicans to search for dispersed food. This assumption is validated by the long periods (77% of the trip time), long distances accumulated (median  = 70 km) and large areas (median  = 119 km^2^) covered during the intermediate commute. With the data available it is uncertain whether pelicans fed during floating or flying bouts. However, Peruvian pelicans may rely on a sit-and wait strategy while feeding on the surface at night [36], [37]. The fact that the floating bouts showed a sinuous displacement rather than a smooth path typical of birds passively drifting with currents [38] suggest that pelicans were actively searching for food while sitting on the water. It is unknown whether Peruvian pelicans are able to detect fish by tactile exploration using the sensitive bill-tip [8], [10] or use bioluminescence generated by planktonic organisms [39]. Peruvian anchovies do not have bioluminescent organs [39], but pelicans may be able to indirectly locate anchovies by following ephemeral bioluminescent tracks that glow when plankton swarms are disturbed by feeding anchovies [40]. Furthermore, prey detection may also be enhanced during bright moonlit nights. Albatrosses and shearwaters from other latitudes are more active at sea during full moons [36], [41], [42], indicating that prey are more readily perceived using visual cues at these times [36]. In this study, pelicans were tracked from first-quarter to full-moon phase, that is, during a period of moonlit nights. Given the short tracking period it is not possible to establish a link between the nocturnal foraging of Peruvian pelicans and the occurrence of bright nights. Our observations need to be contrasted in the future with tracking data from pelicans during the entire lunar-cycle to determine whether these birds can also forage in dark nights.

Kleptoparasitism and predation have been suggested as selective pressures that force seabirds to remain at sea during the night [4], [5] but this hypothesis can be ruled out for our study since potential pirates of Peruvian pelicans such as frigatebirds (*Fregatta* spp.) and large gulls (*Larus* spp.) [16], [17] were absent or present in small numbers on Lobos de Tierra. Peruvian pelicans are also scavengers and pirates [16] and they can benefit from fishing activities throughout fish discards as do other seabird species [6], [7], [43]. Pelicans could follow purse seine vessels that operate during the night to catch anchovies close to the sea surface [34]. However, results from a long-term monitoring program in the last decade along the Peruvian coast indicate that net hauling occurred primarily during daytime, with a median net setting time at 10:00 h (Joo, R., pers. comun., results derived from Bertrand et al. 2008 [44] and Joo et al. 2011 [45]), and therefore the nocturnal behavior of Peruvian pelicans was not linked to the activities of the commercial fishery.
